# SGLT2 Inhibitors: The First Endothelial-Protector for Diabetic Nephropathy

**DOI:** 10.3390/jcm14041241

**Published:** 2025-02-13

**Authors:** Davide Viggiano, Rashmi Joshi, Gianmarco Borriello, Giovanna Cacciola, Annalisa Gonnella, Andrea Gigliotti, Michelangelo Nigro, Giuseppe Gigliotti

**Affiliations:** 1 Department Translational Medical Sciences, University of Campania, 80138 Naples, Italy; rashmi.joshi@unicampania.it (R.J.); gianmarco.borriello@unicampania.it (G.B.); giovanna.cacciola.gc@gmail.com (G.C.); 2 Department Nephrology, Eboli Hospital, 84025 Eboli, Italy; annagon@live.it (A.G.); andreagigliotti73@yahoo.it (A.G.); michelangelonigro@hotmail.it (M.N.); g.gigliotti@aslsalerno.it (G.G.)

**Keywords:** SGLT2, diabetic nephropathy, kidney biopsy, endothelial protection, mitochondrial function, ageing-related kidney disease

## Abstract

Sodium-glucose co-transporter type 2 inhibitors (SGLT2i) have emerged as a class of agents relevant for managing diabetic nephropathy and cardiopathy. In a previous report, we noticed that these drugs share, with other drugs with “nephroprotective” effects, the ability to reduce the glomerular filtration rate (GFR), thus suggesting the kidney hemodynamic effect as a proxy for optimal drug dosage. We also noticed that all known nephroprotective drugs exert cardioprotective functions, suggesting the possibility of activities not mediated by the kidney. Finally, we observe that nephroprotective drugs can be grouped according to their effects on hemoglobin levels, thus suggesting their mechanism of action. While the primary mechanism of SGLT2i involves glycosuria and natriuria, growing evidence suggests broader therapeutic effects beyond hemodynamic modulation. Specifically, the evidence that SGLT2 can be expressed in several atypical regions under pathological conditions, supports the possibility that its inhibition has several extratubular effects. Evidence supports the hypothesis that SGLT2i influence mitochondrial function in various cell types affected by diabetes, particularly in the context of diabetic nephropathy. Notably, in SGLT2i-treated patients, the extent of albumin-creatinine ratio (ACR) reduction post-treatment may be correlated with mitochondrial staining intensity in glomerular endothelial cells. This implies that the anti-proteinuric effects of SGLT2i could involve direct actions on glomerular endothelial cell. Our investigation into the role of SGLT2 inhibitors (SGLT2i) in endothelial function suggests that the aberrant expression of SGLT2 in endothelial cells in T2DM would lead to intracellular accumulation of glucose; therefore, SGLT2i are the first type of endothelial protective drugs available today, with potential implications for ageing-related kidney disease. The review reveals two major novel findings: SGLT2 inhibitors are the first known class of endothelial-protective drugs, due to their ability to prevent glucose accumulation in endothelial cells where SGLT2 is aberrantly expressed in Type 2 Diabetes. Additionally, the research demonstrates that SGLT2 inhibitors share a GFR-reducing effect with other nephroprotective drugs, suggesting both a mechanism for optimal drug dosing and potential broader applications in ageing-related kidney disease through their effects on mitochondrial function and glomerular endothelial cells.

## 1. Introduction

Type 2 Diabetes mellitus (T2DM) stands out among metabolic diseases as the most prevalent chronic hyperglycemic condition, affecting 422 million people globally, as of 2023 (WHO data). Despite a large wealth of information, T2DM retains several enigmatic aspects.

All forms of chronic hyperglycemia can lead to organ damage (affecting the brain, kidneys, heart, and eyes). The most parsimonious explanation for the multi-organ complications observed in T2DM is a dysfunction of the vascular bed (both micro- and macrovascular) [1,2], though the dysfunction of the vascular bed represents only one aspect of the intricate pathogenesis of vascular complications in diabetes.

Nowadays, the treatment of T2DM offers new opportunities, apart from the older hypoglycemic drugs (such as sulfonylureas and insulin) which do not appear to give nephroprotective action.

Specifically, the sodium-glucose co-transporter type 2 inhibitors (SGLT2i) and finerenone are indicated for the treatment of diabetic nephropathy; furthermore, GLP-1 receptor agonists have an interesting nephroprotective effect in diabetic nephropathy, though this might be mediated by their strong effect on weight loss, which may pose a difficulty in interpreting the effects and will be a matter of future analysis.

The SGLT2i represent a novel class of nephroprotective and cardioprotective agents that have gained significant attention for their therapeutic potential in both diabetic and non-diabetic populations [3,4,5,6].

These drugs work primarily by inhibiting the SGLT2 protein, which is highly expressed in the proximal tubule cells of the kidneys [7]. SGLT2 is responsible for approximately 90% of glucose reabsorption in the kidney, making it a crucial target for glucose homeostasis [8]. Therefore, SGLT2i reduce kidney glucose reabsorption, thereby lowering blood glucose levels and exerting beneficial effects on renal and cardiovascular outcomes in T2DM [9].

These antidiabetic drugs demonstrate diverse beneficial effects on hematological and biochemical parameters [10] and lower blood glucose levels [11]. SGLT2i have shown modest modifications of plasma lipid profile. A meta-analysis of 48 randomized controlled trials found that SGLT2 inhibitors significantly increase total cholesterol, LDL-cholesterol, non-HDL-cholesterol, and HDL-cholesterol, while decreasing triglyceride levels [12]. Data from the literature also suggest that SGLT2 inhibitors may reduce liver stiffness (indexed by elastometry) [13] contrasting the increased stiffness observed in T2DM [14], which parallels the decreased elasticity of arteries, particularly when albuminuria is present [15].

## 2. Pathophysiology of Micro- and Macrovascular Complications

Endothelial dysfunction is only one of several important pathophysiological changes that drive the development of T2DM and its complications. These complications arise from a multifaceted interplay of factors, including (i) endothelial dysfunction (e.g., increased blood pressure, increased shear stress, impaired autoregulation), (ii) chronic inflammation (elevated pro-inflammatory cytokines and adhesion molecules), (iii) progressive fibrosis (driven by TGF-β and excessive extracellular matrix deposition), (iv) epigenetic modifications (e.g., DNA methylation and histone modifications), and metabolic derangements (e.g., glycation end-products formation). These processes increase the risk of both microvascular and macrovascular complications [16].

Apart from microvascular complications, macrovascular complications are also frequent. Key macrovascular complications in T2DM consist in the formation of atherosclerotic lesions in regions such as: (i) coronary arteries, increasing the risk of myocardial ischemic events; (ii) cerebral arteries, with risk of ischemic strokes and transient ischemic attacks; (iii) peripheral arteries, particularly in the lower extremities, resulting in claudication and non-healing ulcers [17]. At vascular level, obstructive, non-calcified and distal atherosclerotic plaques [18] are mostly frequent. These macrovascular changes are accompanied by higher risk of thrombosis as compared to the lesions in hypercholesterolemia, hypertension and ageing, which parallel the differential inflammatory levels of these conditions (low in ageing, etc., and high in T2DM). Overall these plaques are more consistent with those in advanced kidney disease and smoking [19].

The Diabetes Control and Complications Trial (DCCT) and its observational follow-up study, the Epidemiology of Diabetes Interventions and Complications (EDIC) [20], along with the United Kingdom Prospective Diabetes Study (UKPDS) [21], provided compelling evidence for the sustained benefits of early intensive glycemic control. These landmark trials demonstrated that early glycemic intervention confers long-term protective effects against both micro- and macrovascular complications, a phenomenon termed the ’legacy effect’ or ’metabolic memory’. The molecular basis for this phenomenon has been attributed to persistent epigenetic modifications induced by prior hyperglycemic episodes. Specifically, these epigenetic alterations involve changes in DNA methylation patterns, histone modifications, and non-coding RNA expression, which collectively influence the transcriptional regulation of genes involved in diabetic complications [22,23].

### T2DM as a Disease of Endothelial Basement Membrane

T2DM is characterized by dyslipidemia, and, at kidney level, tubular hypertrophy which manifests in kidney hypertrophy [24,25,26], proteinuria and initial hyperfiltration [27] which evolve towards reduced eGFR [28].

T2DM presents mostly in obese [27], aged, and hypertensive (mostly systolic) subjects [29,30]; biochemically T2DM shows normal or high levels of C-peptide [31], insulin and amylin [32], increased plasma amino acids [33], decreased level of growth hormone [34,35]. Histologically glomerulomegaly with rare Kimmelstiel–Wilson nodules [36] are reported.

**Figure 1:**
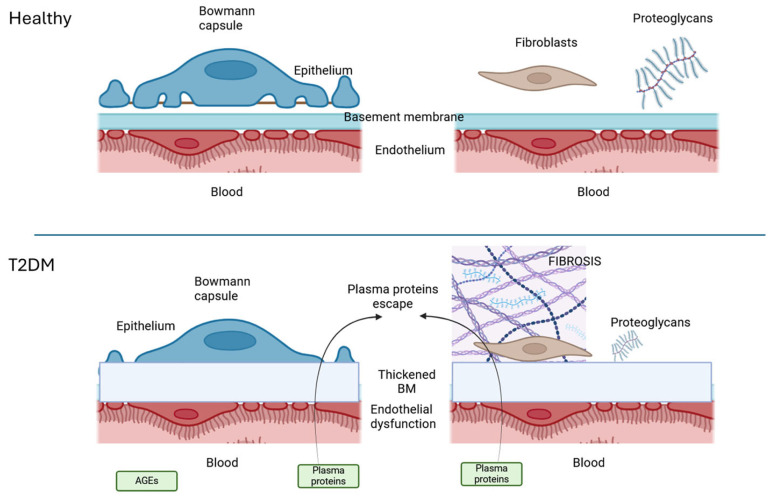
T2DM as a disease of basement membranes (BM). The alteration of endothelial cells induced by advanced glycated products (AGEs) in T2DM is then responsible for the modified composition of the BM. In the case of the glomeruli (left) this causes proteinuria. In the case of other organs the resulting plasma protein escape induces fibrosis and the typical macro and microvascular damages of T2DM (image partially prepared with Biorender).

The basement membrane (BM) is produced by a cooperative effort of connective cells and epithelial cells, such as endothelial cells and tubular cells. Notably, only in few regions of the body, two closely facing epithelia contribute to BM synthesis without connective interposition, such as in the kidney glomeruli (see Figure 1). Thus, the BM represents the extreme barrier from the blood and its alteration inevitably produces the escape of plasma proteins in the behind-the-endothelial tissue, whether it is an alveolus, or a Bowmann space, or an interstitial connective tissue. It is only in the latter that enduring alterations can be observed, as the escape of plasma material [19] cannot be drained off easily: the interstitial proteoglycans—critical for regulating hydration, tissue architecture, and signalling—are therefore severely disrupted in T2DM [37], exacerbating extracellular matrix instability and promoting fibrosis [38].

Therefore, T2DM can be considered a disease of BM. Due to the similarity with changes occurring in ageing, it may also be recognized as a model of accelerated ageing [39], particularly through its effects on the extracellular matrix and basement membranes throughout the body [40].

A hallmark feature of T2DM, proteinuria underscores systemic basement membrane (BM) instability [41], across various tissues, a systemic breakdown in tissue barriers that closely parallels ageing-related deterioration. This allows the escaping of plasma proteins into the interstitium in T2DM (see Figure 1). The gel-to-sol transition—a shift in the extracellular matrix’s physical state—further destabilizes tissues by increasing interstitial volume, triggering fibroblast activation, and promoting fibrosis.

In T2DM the BM are thickened [42] with an altered composition and reduced functional integrity. These changes mimic the progressive degeneration seen in normal ageing.

Adding to these challenges is the dysregulation of the growth hormone (GH) and insulin-like growth factor-1 (IGF-1) axis in T2DM, a key regulator of tissue repair and cellular turnover. In ageing, a natural decline in GH/IGF-1 signalling slows regeneration and contributes to metabolic inefficiencies [43]. In T2DM, this axis faces additional perturbations due to hyperinsulinemia and insulin resistance, compounding tissue damage and accelerating the ageing-like features of diabetic nephropathy. External factors such as circadian fluctuations in leg extracellular volumes, where prolonged standing and diminished lymphatic drainage create chronic low-grade triggers for tissue remodelling, mirror mechanisms seen in ageing. Hemodialysis exacerbates this dynamic instability by causing abrupt fluid shifts that strain extracellular compartments, highlighting the delicate balance required to maintain homeostasis [44].

Key drivers of fibrosis, such as elevated venous pressure and impaired lymphatic drainage, underscore the interconnectedness of vascular and lymphatic systems in controlling tissue health. Elevated venous pressure contributes to interstitial edema, while lymphatic dysfunction limits the clearance of proteins and waste products from tissues.

Overall, this simplified model of T2DM explains why the key issue in this disease, as well in other diseases with modifications of blood composition, is endothelial dysfunction.

## 3. What Is a Drug with Nephroprotective Effect and When Is It Cardioprotective?

A drug can be called “nephroprotective” when it slows down the natural progression of kidney function disease, irrespective of the cause. This definition excludes the immunosuppressive and steroidal drugs from the set of “drugs with nephroprotective effect”, as they can decrease or halt kidney damage only in specific cases of glomerulonephritis, whereas are not commonly employed in other causes of kidney failure. The notion of “nephroprotection” is tightly linked to the definition of “chronic kidney disease” (CKD) as an umbrella term of all diseases of the kidney with a reduction of the glomerular filtration rate (GFR) lasting longer than three months, introduced by the K/DOQI group in 2002 [45]. After the historical K/DOQI classification, the term CKD was retained in the new classification system of KDIGO [46]. The term “chronic kidney disease” (CKD) gained widespread adoption in medical practice during the 1990s, coinciding with clinical trials that demonstrated angiotensin-converting enzyme (ACE) inhibitors could significantly slow the progression of kidney damage, irrespective of the cause. Actually, the first demonstration of nephroprotective effect of ACE inhibitors (in 1993) was limited to the case of diabetic nephropathy [47]. It was only four years later, in 1997, that ACE inhibitors were shown to work in non-diabetic nephropathies [48], the same dynamic of discovery occurring nowadays with SGLT2i.

At present, few drugs with nephroprotective effects are available: ACE inhibitors (and sartans or RASi: Renin-Angiotensin System inhibitors), SGLT2i, finerenone [49], and, limited to the case of adult polycystic kidney disease (ADPKD), vaptans [50,51]. As proposed by Viggiano et al. [52], all these drugs share a similar phenomenon: paradoxical reduction of the GFR at the beginning of the treatment. Therefore, the authors have suggested that the major characteristic of a nephroprotective drug should reside in its ability to reversibly reduce the GFR.

All these drugs also have in common the fact that have been tested first in T2DM, before showing they were effective without T2DM (see also Table 1). Subsequent evidence indicated nephroprotective benefits in individuals with proteinuria, extending beyond their use in diabetic populations.

Notably all of these nephroprotective drugs also show cardioprotective efficacy (usually indexed by a reduction in cardiovascular death or heart failure hospitalization), even in absence of kidney disease. Specifically, SGLT2i [53], as well as ACE inhibitors [54] reduce CV risk in HF reduced ejection fraction. Similarly, finerenone reduces cardiovascular (CV) risk in all patients with heart failure (HF). Finerenone is a non-steroidal mineralocorticoid antagonist (nsMRA), in contrast with steroidal mineralocorticoid antagonists (sMRA), which are active only in the presence of a reduced ejection fraction [55]. Indeed, sMRA (e.g., spironolactone, eplerenone) are available since long time, but their steroidal structure gives more side effects compared to finerenone (gynecomastia, hyperkalemia); furthermore, finerenone has superior benefits than other MRA for diabetic kidney disease.

An interesting hypothesis about the non-renal effects of SGLT2i suggests a relevant role of changes in the metabolism such as (i) glucose/fatty acids to ketones metabolic shift in myocardium, (ii) weight loss [56] (iii) improved plasma lipid profile (iv) reduction in blood pressure [56]. These pleiotropic effects cannot be underestimated, though their relevance for renal protection is unclear.

Indeed, a comprehensive meta-analysis (nearly 100,000 patients), found a mean weight reduction of 1.79 kg in subjects using SGLT2i compared to placebo, including patients without T2DM [57]. Accordingly, SGLT2i appear to reduce epicardial fat thickness by 20%, though this effect was studied in diabetic patients [58]. Finally, patients with IgA glomerulopathy appear to benefit of SGLT2i treatment [59]. Therefore, it is possible that the endothelial protection may also pertain to conditions without T2DM, such as obesity and glomerulopathies of non-diabetic origin.

Though not systematically tested, it is possible that the effect of these drugs is addictive.

A major exception in the group of drugs with nephroprotective effect is represented by vaptans, which may ameliorate acute HF symptoms, but find no benefits in long term treatments of HF [60].

An additional class of potentially nephroprotective agents are the combination of angiotensin receptor-nephrilysin inhibitor (ARNI), such as sacubitril/valsartan. While they have proven effects on HF [61], their effectiveness as drugs with nephroprotective effects awaits further confirmation, though they may reduce proteinuria [62]. As for the other drugs with nephroprotective effect, they also increase creatinine levels (reduce eGFR) [63] and reduce hemoglobin as the RAASi [64].

Although the kidney and heart are linked in health and disease, it is unclear whether this is more a result of sharing the same fate (e.g., a common damage to small vessels) or their interdependence, particularly when nephro-cardio-protective drugs are used. In the following analysis, we will consider the possibility that the positive multiorgan effects of SGLT2 are mediated by endothelial cells, although this clearly does not exclude that the renal hemodynamic effects may also have positive implications at the cardiac level. One reason why this last possibility has limited explanatory value is that the cardioprotective effects of SGLT2i (as well as RASi and MRA) can also be observed in the dialysis patient (without a working kidney). Specifically, the DAPA-CKD trial included patients with advanced CKD (eGFR < 30 mL/min/1.73 m²), suggesting renoprotective and cardioprotective effects, even in advanced CKD [65]. Similarly, RASi also have protective effects on heart and kidney in hemodialysis patients [66]. In contrast, there is no available data about finerenone in hemodialysis; however spironolactone, an sMRA, reduces cardiovascular mortality in hemodialysis patients [67].

## 4. Effects of Drugs with Nephroprotective Effect on Hemoglobin

Anemia, common in diabetic nephropathy, contributes to endothelial dysfunction by reducing oxygen delivery, increasing oxidative stress, and promoting inflammation. Drugs with nephroprotective effect can influence hemoglobin levels, which in turn affects endothelial function. The change in hemoglobin impinges upon endothelial function through the levels of nitric oxide, oxidative stress, and pro-inflammatory cytokine activity. Thus, the effects of drugs with nephroprotective effect on hemoglobin levels can either mitigate or aggravate endothelial dysfunction, depending on the mechanism of action.

Drugs with nephroprotective effect, while pivotal in managing kidney health, share a common impact: all known drugs in this category—RAAS inhibitors, vaptans, finerenone, and SGLT2 inhibitors—reduce the glomerular filtration rate (GFR) [52]. Despite this shared trait, these drugs exhibit markedly different effects on hemoglobin levels, driven by varying interactions with angiotensin II (AT-II).

**Figure 2:**
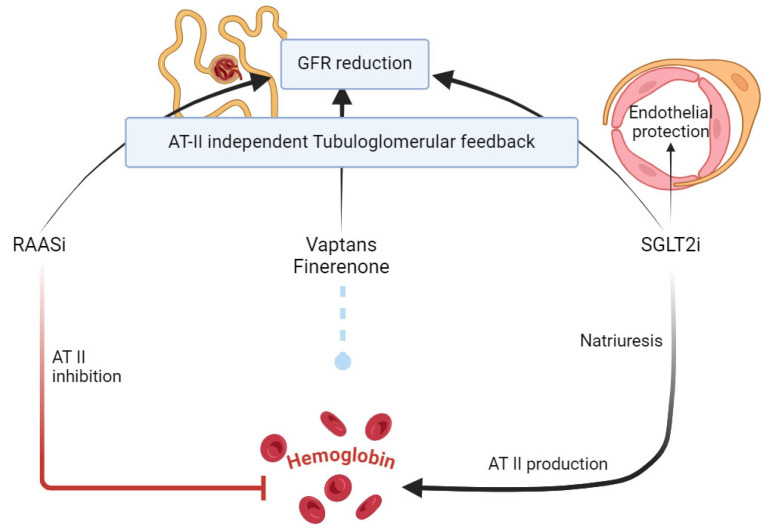
Effects of SGLT2i and other drugs with nephroprotective effect on hemoglobin.

Angiotensin II plays a dual role, not only in renal hemodynamics but also in stimulating erythropoiesis [68]. As illustrated in Figure 2, RAAS inhibitors (RAASi) may reduce hemoglobin levels through attenuating angiotensin II-mediated erythropoiesis. This reduction in erythropoiesis underscores the hematological side effects associated with these agents [10].

In contrast, SGLT2 inhibitors (SGLT2i) remain distinct. These drugs increase hemoglobin levels, potentially through an opposing mechanism: their induced natriuresis triggers a compensatory rise in AT-II production. This unique interplay between natriuresis and erythropoiesis highlights a distinct advantage of SGLT2i over other drugs with nephroprotective effect.

SGLT2i are reported to target different routes to regulate the blood glucose and erythropoiesis [10]. Indeed, they are known to increase hemoglobin production [69]. The pathway through which this happens remains unclear (see Figure 2).

At variance, finerenone and vaptans appear to exert a negligible influence on AT-II activity. Consequently, these agents do not significantly alter hemoglobin levels, offering a neutral hematological profile [70].

The conclusion is that drugs with nephroprotective effects reduce GFR through an AT-II independent tubuloglomerular feedback mechanism. Among these, only SGLT2 inhibitors provide additional endothelial protective benefits, further differentiating them within this therapeutic class.

This nuanced understanding of drugs with nephroprotective effects on GFR and hemoglobin offers a roadmap for optimizing treatment strategies, balancing renal and systemic outcomes.

## 5. Off-Target Effects of SGLT2 Inhibitors

Emerging evidence suggests that the benefits of SGLT2i may extend beyond their hemodynamic effects.

Recent studies showed that SGLT2i are important in enhancing vitality and shielding endothelial cells from Advanced Glycated End Products (AGEs), by reducing the latter [71]. These should be considered as a general stressor for endothelial cells. Indeed, an analysis on endothelial cells in culture has shown that, in the presence of AGEs, cells reduce their vitality, an effect that was reversed by SGLT2i. Endothelial senescence is believed to be linked to vascular function; therefore, it is necessary to study the effect of SGLT2i on such non-renal tissues to better understand the mechanism behind kidney diseases [72].

SGLT2i appear to favour the metabolism of fatty acids and ketone bodies in the skeletal muscle [73], which may be relevant for the differentiation of skeletal muscles [74].

Though the renal expression of SGLT2 is known to be limited to the proximal tubule of the kidney in healthy conditions [75], under non-physiological conditions extrarenal expression can be observed. Specifically pathological conditions such as liver disease can lead to the expression of SGLT2 in the liver, where it is usually not detectable [76].

In diabetic patients, chronic hyperglycemia leads to the over-expression of SGLT2, not only in renal proximal tubule cells but also in other cell types, including endothelial cells [77,78], mesangial cells [79], and cardiomyocytes [7,80,81].

This broad expression pattern implies that SGLT2 inhibitors could exert protective effects in these non-renal tissues as well. The expression of SGLT2 in these non-renal tissues is thought to be a maladaptive response to chronic hyperglycemia, potentially contributing to organ dysfunction in T2DM [78,82,83,84].

**Figure 3:**
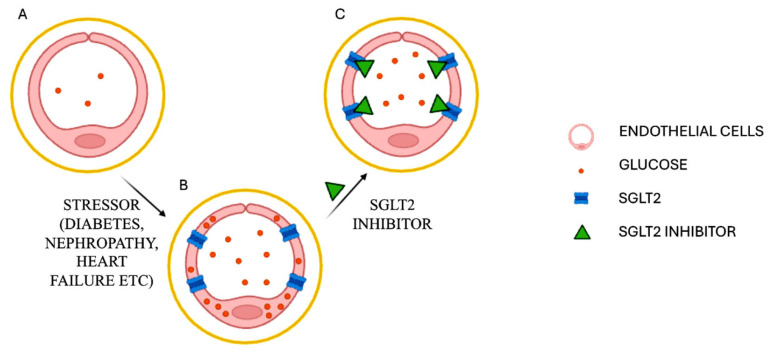
A conceptual model to explain the efficacy of SGLT2i on endothelial cells A. Normal endothelial cells do not express SGLT2; B. in the presence of stressors the endothelial cells express SGLT2 protein, leading to intracellular accumulation of glucose and Na+ and consequent endothelial dysfunction; C. SGLT2 inhibitors, by inactivating SGLT2 protein, alleviate the accumulation of glucose and Na+ inside endothelial cells.

The expression of SGLT2 on endothelial cells under stress conditions strongly suggests that SGLT2i can act through a unique mechanism of action, described in Figure 3. Specifically, the aberrant expression of SGLT2 in endothelial cells in T2DM or other stresses would lead to an intracellular accumulation of glucose and sodium. This unphysiological change in the internal cellular environment and of its volume may represent a relevant mechanism of endothelial dysfunction. SGLT2i, by blocking this event, provide endothelial protection. In this scenario, they represent the first type of endothelial protective drugs available today.

As suggested in Figure 3, the aberrant expression of SGLT2 in endothelial cells in T2DM or other stresses would lead to intracellular accumulation of glucose: this may clearly explain why SGLT2i are the first type of endothelial protective drugs available today.

## 6. SGLT2i Directly Influence Mitochondrial Function?

Since mitochondrial dysfunction is a key contributor to the pathogenesis of both diabetic nephropathy and cardiomyopathy, the ability of SGLT2 inhibitors to modulate mitochondrial function could be a critical mechanism underlying their protective effects [85]. Mitochondrial dysfunction in T2DM is characterized by impaired oxidative phosphorylation, increased ROS production, and altered mitochondrial dynamics (fusion and fission) [86,87]. These changes can lead to cellular energy deficits, oxidative stress, and activation of pro-inflammatory and pro-fibrotic pathways [88].

Mitochondrial volume regulation depends on inner membrane permeability and osmotic balance, which are influenced by various electrolytes [89] and potentially by intracellular glucose levels through SGLTs. Recent evidence suggests that SGLT inhibitors affect mitochondrial dynamics across multiple cell types by modulating intracellular sodium and hydrogen ion levels. While the exact mechanisms linking SGLTs to mitochondrial ion channels remain poorly understood, growing evidence of SGLT2i effects on mitochondrial function is driving further research in this field [82].

SGLT2 inhibitors alter the intracellular concentrations of sodium and glucose, which are critical regulators of various cellular processes [90]. These changes could have downstream effects on mitochondrial function, as mitochondria are highly sensitive to fluctuations in ionic and metabolic environments [91]. For example, shifts in intracellular sodium and glucose levels may influence mitochondrial swelling [92], bioenergetic activity, and the production of reactive oxygen species (ROS).

The sodium-calcium exchanger (NCX) in the mitochondrial membrane is particularly sensitive to changes in intracellular sodium, and its activity can significantly impact mitochondrial calcium levels and, consequently, energy production and ROS generation [82,93,94,95].

However, recent data suggest that the anti-proteinuric effects of SGLT2i may also involve direct actions on the mitochondria of endothelial cells within the glomeruli [96].

Mitochondrial dysfunction in endothelial cells is a key factor in the pathogenesis of diabetic nephropathy, leading to increased oxidative stress, inflammation, and cellular apoptosis, all of which can exacerbate endothelial damage and contribute to proteinuria [97]. Specifically, mitochondrial dysfunction can lead to increased ROS production [98], impaired ATP production in endothelial cells [99], disrupted calcium homeostasis [100], cytochrome c release with activation of apoptosis [100].

SGLT2i, by altering intracellular sodium levels, may influence the activity of the sodium-calcium exchanger (NCX) on the mitochondrial membrane, potentially impacting mitochondrial calcium homeostasis [101,102]. The mitochondrial NCX (mNCX) plays a crucial role in extruding calcium from the mitochondrial matrix in exchange for sodium ions [94,95,103,104,105]. Calcium plays a pivotal role in mitochondrial bioenergetics, influencing the activity of key enzymes in the tricarboxylic acid (TCA) cycle, such as α-ketoglutarate dehydrogenase and isocitrate dehydrogenase, and the electron transport chain [106]. Disruptions in mitochondrial calcium levels can lead to impaired ATP production, increased production of reactive oxygen species (ROS), and eventually, mitochondrial dysfunction [107]. Furthermore, changes in intracellular sodium concentrations could affect the activity of the mitochondrial Na+/H+ exchanger (mNHE), which regulates mitochondrial matrix pH [108]. Alterations in matrix pH can influence the activity of various mitochondrial enzymes and impact overall mitochondrial function [109]. The potential for SGLT2i to modulate mitochondrial function via sodium-dependent mechanisms could provide a novel explanation for their protective effects against proteinuria, particularly in the context of diabetic nephropathy.

The proportional relationship between SGLT2i-induced mitochondrial effects in glomerular endothelial cells and their anti-proteinuric action further supports the notion that these drugs may exert their renal protective effects through multiple pathways, including those involving mitochondrial health.

## 7. Blood Pressure and Mitochondrial Activity in Proximal Tubule Cells

The blood pressure is tightly controlled by the kidney, with proximal tubular cells exerting a pivotal role in this process [110].

Consistently in animal models, it is known that proximal tubule cells consume oxygen at a higher rate in hypertensive animals, possibly mediated by greater pyruvate dehydrogenase complex activity [111]. Notably, mitochondria in proximal tubules appear to express a specific receptor for Angiotensin II/AT1aR), which could increase oxidative and glycolytic responses and elevates blood pressure mediated by superoxide/NHE3 (the Na^+^/H^+^ exchanger 3) [112]. Proximal tubular cells are provided with connexins, which, similar to what happens in the brain [113], are pivotal for their correct functioning.

## 8. Diabetic Albuminuria and Mitochondria in Endothelial Cells

Proteinuria and albuminuria, characterized by the presence of excess albumin in the urine, is a common complication in diabetic patients and serves as an important marker of kidney damage and a predictor of cardiovascular risk [40,114]. It is usually indexed by the albumin to creatinine ratio in the urine (ACR).

The development of proteinuria in T2DM is primarily driven by damage to both the endothelial cells lining the glomerular capillaries and the podocytes, which are specialized cells that form a crucial part of the glomerular filtration barrier [115].

In a healthy kidney, the glomerular filtration barrier, consisting of fenestrated endothelium, glomerular basement membrane (GBM), and podocyte foot processes with interposed slit diaphragms, prevents the passage of large molecules such as proteins into the urine.

However, in diabetic nephropathy, hyperglycemia and related metabolic disturbances lead to structural and functional damage in these cell compartments, compromising the integrity of the filtration barrier and resulting in proteinuria [40].

The pathophysiology of diabetic nephropathy involves several interconnected mechanisms resulting in podocyte injury and fibrosis [116].

SGLT2i have demonstrated a capacity to reduce proteinuria in diabetic patients [117]. This effect was traditionally attributed to their hemodynamic actions. By inhibiting sodium-glucose co-transporters in the proximal tubules, SGLT2i reduce glucose reabsorption and promote natriuresis (excretion of sodium in the urine) [118]. This leads to a decrease in intraglomerular pressure, thereby alleviating the glomerular hyperfiltration that contributes to proteinuria [119].

Though the tubule-glomerular feedback mechanism plays a role in this process [51,52,120,121,122], recent data suggest that the anti-proteinuric effects of SGLT2i may also involve direct actions on the mitochondria of endothelial cells within the glomeruli [96,123,124].

**Figure 4:**
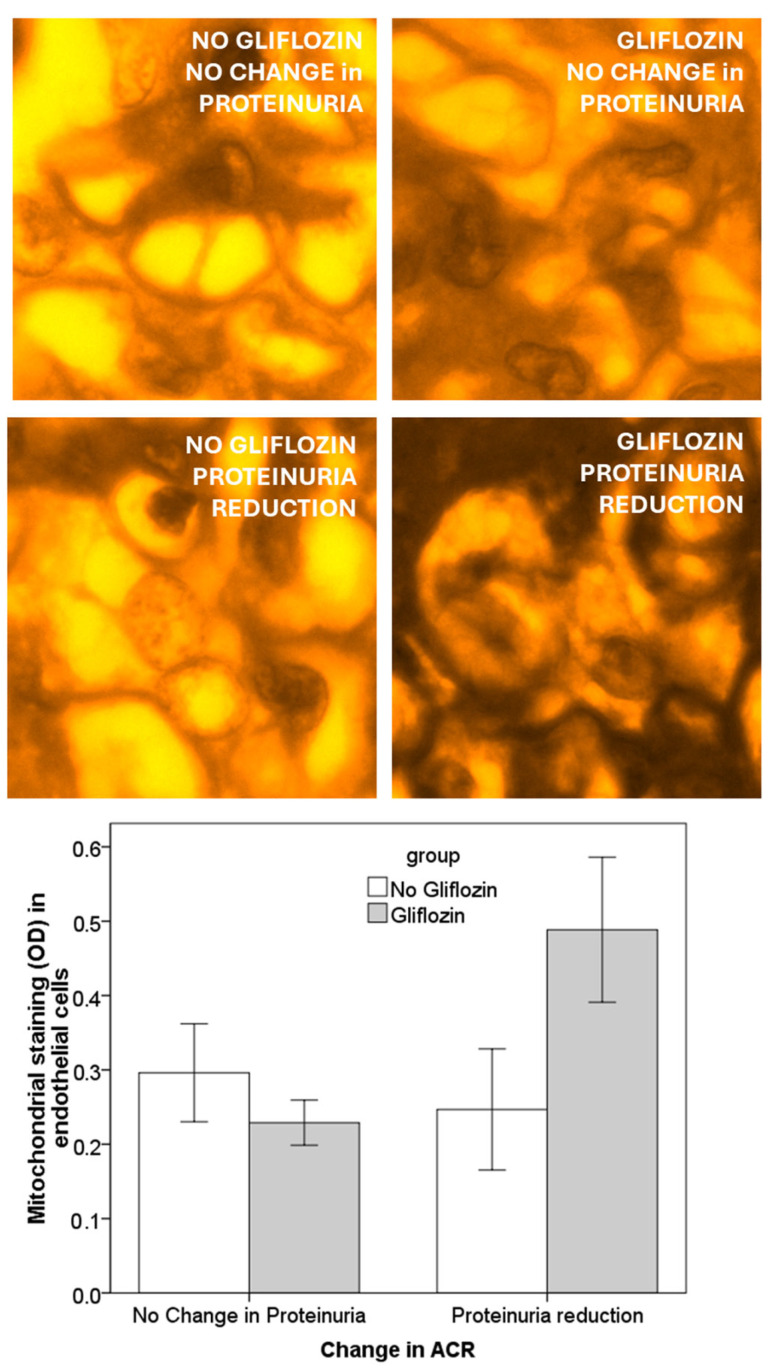
Relationship between proteinuria (ACR: albumin creatinine ratio) and mitochondrial density in endothelial cells of kidney biopsies. The left panel refers to patients without SGLT2i, whereas right panel describes the biopsies of SGLT2i treated patients. Treatment with SGLT2i modifies the relationship between endothelial mitochondrial activity and proteinuria.

In a preliminary report, we have analyzed kidney biopsies from diabetic patients in treatment with a SGLT2i (*n* = 10) or without SGLT2i treatment (*n* = 10), stained with fuchsin red, which gives a colour intensity proportional to mitochondrial content [96]. Clinical variables (albumin-creatinine ratio (ACR), blood pressure, eGFR, glycated albumin) have been measured before and after 4 months of treatment. In diabetic patients without SGLT2i, the mitochondrial content of endothelial cells was not related to the extent of ACR reduction over time. In diabetic patients treated with SGLT2i, the extent of ACR reduction after treatment was correlated with the intensity of mitochondrial staining in glomerular endothelial cells (Figure 4). SGLT2i treatment may induce a change in the mitochondrial content of endothelial cells, particularly in patients responding with a reduction in proteinuria. 

The role of SGLT2i in endothelial function highlighted their protective effects, ameliorating endothelial dysfunction and improving cell vitality (see also recent review [125]).

## 9. Conclusions

Several limitations exist that warrant careful consideration. First, uncertainty exists about nephroprotective effects in patients with earlier stages of diabetic nephropathy.

Another notable gap lies in understanding the effects of SGLT2i on endothelial cells and microvascular function. While preclinical studies suggest that SGLT2i may improve endothelial health, by reducing oxidative stress, inflammation, and vascular stiffness, these effects have not been robustly confirmed in human studies. The available data are mostly indirect, inferred from improvements in macrovascular outcomes and reductions in markers like albuminuria. Furthermore, the interplay between SGLT2 inhibition and endothelial repair mechanisms, such as angiogenesis or endothelial progenitor cell function, remains largely unexplored. Given the critical role of endothelial cells in maintaining glomerular integrity and function, this represents a significant knowledge gap.

Future research should address these limitations by conducting studies that specifically evaluate SGLT2i in early stages of diabetic nephropathy. Dedicated trials focusing on the direct effects of SGLT2i on endothelial biomarkers, vascular imaging, and histological studies in kidney tissue are also necessary to elucidate their role in endothelial health.

Further research is warranted to explore the exact molecular mechanisms by which SGLT2i influence endothelial function and to determine how these effects contribute to their overall therapeutic efficacy in diabetic kidney disease.

Although new methods are being developed to interrogate proteinuria and the effects of SGLT2i, understanding these mechanisms mentioned above could lead to the development of more targeted therapies for diabetic nephropathy and other kidney diseases characterized by mitochondrial dysfunction and proteinuria.

Finally, the exploration of endothelial dysfunction and ageing led us to propose a link between endothelial senescence and vascular function, with potential implications for ageing-related kidney disease.

## Figures and Tables

**Table 1:** Differences in efficacy, mechanisms, and clinical outcomes between SGLT2i and other nephroprotective drugs. ↔: no change; ↓: decrease; ↑: increase; RASi: Renin-Angiotensin System inhibitors.

	SGLT2i	Finerenone	RASi
Efficacy	HbA1c: ↓~0.5–1% Proteinuria: ↓~30–50% eGFR: ↓~3–5 mL/min BP: ↓~4–5 mmHg urinary Na+: ↑ plasma K+: ↔	HbA1c: ↔ Proteinuria: ↓~30% eGFR: ↓ 2–4 mL/min BP: ↓~3–5 mmHg urinary Na+: ↔ plasma K+: ↑	HbA1c: ↔ Proteinuria: ↓~30–50% eGFR: ↓~10–20% BP: ↓ 10–20 mmHg urinary Na+: ↔ plasma K+: ↑
Mechanisms	Inhibit SGLT2 in renal proximal tubule and in stressed endothelial cells Metabolic effects	Inhibit mineralocorticoid receptor, reducing inflammation and fibrosis	Inhibit angiotensin II production or its receptor
Clinical outcomes	Slower progression of CKD, improved CV mortality in T2DM, Reduce heart failure hospitalization by ~30%	Slower progression of CKD and cardiovascular disease in diabetic patients; reduced heart failure hospitalizations	Slower CKD progression, reduced CV events, better survival in heart failure.
